# Prohibitin (PHB) expression is associated with aggressiveness in DLBCL and flavagline-mediated inhibition of cytoplasmic PHB functions induces anti-tumor effects

**DOI:** 10.1186/s13046-019-1440-4

**Published:** 2019-11-04

**Authors:** Hafidha Bentayeb, Marine Aitamer, Barbara Petit, Lydie Dubanet, Sabria Elderwish, Laurent Désaubry, Armand de Gramont, Eric Raymond, Agnès Olivrie, Julie Abraham, Marie-Odile Jauberteau, Danielle Troutaud

**Affiliations:** 1 EA3842, Université de Limoges, Limoges, France; 2 Laboratoire d’Anatomie-Pathologique, CHU de Limoges, Limoges, France; 3 UMR 7203, CNRS - Université Paris Sorbonne, Paris, France; 4 Sino-French Joint Lab of Food Nutrition/Safety and Medicinal Chemistry, College of Biotechnology, Tianjin University of Science and Technology, Tianjin, 300457 China; 5 AFR Oncology, 1 place Paul Verlaine, Boulogne-Billancourt, France; 6 Groupe Hospitalier Saint-Joseph, Paris, France; 7 Service d’Hématologie Clinique, CHU de Limoges, Limoges, France; 8 Service d’Immunologie, CHU Limoges, Limoges, France

**Keywords:** DLBCL, PHB, FL3, ERK signaling, Akt, Apoptosis

## Abstract

**Background:**

Diffuse large B-cell lymphomas (DLBCLs) are aggressive lymphomas accounting for approximately a third of non-Hodgkin lymphomas. Prohibitin 1 (PHB1) and prohibitin 2 (PHB2) are scaffold proteins that promote mitochondria homeostasis and consequently cell survival, but biological functions of cytoplasmic PHBs remain largely unknown in DLBCL.

**Methods:**

PHB expression was analyzed in 82 DLBCL biopsies and five DLBCL cell lines by immunohistochemistry (IHC) and Western blotting. Pharmacological inhibition of PHB using the synthetic flavagline FL3 was realized in vitro to gain insight PHB cellular functions. Effects of FL3 on DLBCL cell line viability, apoptosis, C-Raf-ERK–MNK–eIF4E signaling pathway and eIF4F complex formation and activity were evaluated by XTT assay, annexin V-FITC/PI dual staining and Western blotting respectively. Subcutaneous DLBCL xenograft model in SCID mice was also performed to determine in vivo FL3 effect.

**Results:**

As in DLBCL cell lines, PHB1 and PHB2 were expressed in germinal center B-cell–like (GCB) and activated B-cell–like (ABC) subtypes. In patient samples, high PHB levels were associated with higher serum LDH (PHB1 and PHB2), IPIaa (PHB2), and Ki-67 (PHB2) expression. Higher PHB1 expression tends to be associated with shorter event-free survival (EFS) in patients, especially in male patients. FL3 induced apoptosis of DLBCL cell lines that was associated with inhibition of the ERK-MNK-eIF4E signaling pathway, including aggressive double/triple-hit DLBCL cell lines. This resulted in altered eIF4F complex formation and activity leading to a reduction of Bcl-2 and c-Myc expression levels. Moreover, FL3 strongly downregulated DLBCL cellular levels of Akt protein and *AKT* mRNA. FL3 antitumor activity was also confirmed in vivo in a murine xenograft model.

**Conclusion:**

Our data indicate that PHB overexpression is associated with markers of tumor aggressiveness in DLBCL, and that targeting PHBs may be a therapeutic option, notably in aggressive subtypes.

## Background

Diffuse large B-cell lymphomas (DLBCLs) are highly aggressive non-Hodgkin lymphomas for which the incidence increases with age (median age at diagnosis is 70 years). Gene expression profiling subdivides DLBCLs into two major subtypes, the germinal center B-cell (GCB) and activated B-cell (ABC) subtypes. Among them, patients with GCB DLBCL have a better prognosis [1, 2]. R-CHOP immunochemotherapy (a combination of anti-CD20 antibody, rituximab, with CHOP chemotherapy) provides a significant survival benefit to most patients, but approximately 15–25% of patients exhibit primary refractory disease and 20–30% relapse after initial response to therapy [3]. Furthermore, double-hit (DHL) and more rarely triple-hit (THL) lymphomas are subgroups of very aggressive lymphomas with both *MYC* and *BCL-2* and/or *BCL6* gene rearrangements characterized by a rapidly progressing clinical course that is refractory to treatment and poor outcome after standard R-CHOP therapy. Thus, these groups of patients represent a huge therapeutic challenge [4, 5].

Different mechanisms in each DLBCL subtype can activate the PI3K/ Akt/ mTOR pathway to enhance cellular growth and metabolism in DLBCL [6]. In the GCB subtype, the loss of PTEN protein expression correlates with PI3K/Akt/mTOR activation [7]. In contrast, constitutive phosphorylation of Akt was not related with loss of PTEN in ABC DLBCL. The hallmark of the ABC subgroup of DLBCL is the constitutive activation of the nuclear factor κB (NF-κB), which promotes cell survival, proliferation and inhibition of apoptosis. This is largely due to the constitutive activation of the “CBM” signaling complex (formed by CARD11, BCL10 and MALT1) [8]. New therapeutic inhibitors directly targeting PI3K/Akt/mTOR pathway have been developed to treat notably relapsed/refractory DLBCL, some of them being currently under investigation in clinical trials [6, 9].

Several reviews have described the potential importance of therapies that target protein translation in cancer including DLBCL [10, 11]. The translation initiation factor 4F (eIF4F) complex, an important downstream target of the mTOR pathway, plays a critical role in the regulation of cap-dependent translation of mRNAs that mostly encode proteins involved in cellular growth, angiogenesis, survival, and malignancy (e.g. cyclin D1, c-Myc, VEGF, and Bcl-2) [12]. This complex contains a translation initiation factor 4E (eIF4E), a scaffolding protein eIF4G, and the RNA helicase eIF4A. eIF4E has been implicated in tumorigenesis, including lymphomagenesis, and eIF4E phosphorylation upon MNK1/2 activation is required for its oncogenic role [11, 13].

Flavaglines are natural compounds extracted from medicinal plants of the genus *Aglaia* that display potent anticancer activities [14, 15]. These compounds trigger apoptosis through various pathways and inhibit the proliferation of tumor cells at low concentrations without toxicity to normal cells [14–16]. Flavaglines exert their activities notably by binding to prohibitins (PHBs) [17]. Prohibitin 1 (PHB1) and prohibitin 2 (PHB2) are scaffold proteins mainly located in the mitochondria, nucleus and plasma membrane, that elicit multiple functions according to their cellular localization and cell type. These functions include nuclear transcription, plasma membrane lipid scaffold protein, mitochondrial morphogenesis and apoptosis [18–21]. Prohibitins have been implicated in cancer progression through regulation of key cell signaling pathways known to induce cell proliferation, resistance and metastasis, including the Ras-mediated c-Raf-MEK-ERK signaling pathway [22, 23]. Binding of flavaglines to PHBs specifically inhibits the PHB-c-Raf interaction, thus inhibiting MEK/ERK/MNK signaling, leading to inhibition of eIF4E phosphorylation. Consequently, flavaglines inhibit the cap-dependent synthesis of many proteins involved in cell division and resistance to apoptosis [17].

PHB protein complex has been recently demonstrated to be required for mitochondrial homeostasis and cell survival included in normal and malignant B cells [24, 25]. However, the clinical significance of PHB expression in DLBCL remains to be determined. Thus, in the present study, we determined the clinical relevance of PHB1 and PHB2 in 82 DLBCL tumor samples and show their potential importance in DLBCL cell survival and proliferation. Furthermore, we also investigated the PHB-mediated cellular signaling pathways in vitro using the PHB-ligand FL3. Our findings on DLBCL cell lines and DLBCL xenograft murine model show PHB involvement in ERK-MNK-eIF4E and Akt pathways and strongly suggest that targeting PHBs using flavaglines may be a potential therapeutic strategy in DLBCL.

## Methods

### Patient samples

Tumor samples were obtained before treatment from 82 patients with de novo DLBCL treated in the hematology department of Dupuytren Hospital (Limoges, France). The clinical features of patients are summarized in Table 1. Tumors were classified according to the World Health Organization classification [26] and assigned as germinal center B-cell (GCB) or non-GCB subtype using the Hans algorithm [27] as previously described [28]. All samples were studied after approval of the local ethics committee (N° 98–2012-23).

**Table 1:** Clinical and immunophenotypical characteristics of DLBCL patients

Features	No. of Patients (%)
Age (years)	62
< 60	31 (38)
≥ 60	51 (62)
Sex Male/Female	48 /34 (59/41)
IPI	
0–2	42 (51)
≥ 3	40 (49)
IPI Age adjusted (IPIaa)	
0	17 (21)
1	27 (33)
2	20 (24)
3	18 (22)
Ann Arbor stage	
I/II	29 (35)
III/IV	53 (65)
Serum lactate dehydrogenase (>1x normal)	41 (51)
Pathological type	
GCB	49 (60)
Non-GCB	33 (40)
Therapy regimen	R-CHOP
Median follow-up time, months	53.7 (0.4–202.6)
5-year OS	65.9
5-year PFS	60.6

### Isolation of normal B cells

For PHBs analysis, peripheral B lymphocytes from 4 healthy volunteer donors were obtained from blood samples. Briefly, B cells were enriched from ficoll-Paque-separated peripheral blood mononuclear cells (PBMC) by negative magnetic cell sorting of as previously reported [29].

### Cell culture and viability assay

Two GCB-DLBCL lines (SUDHL4 and SUDHL6 - DSMZ) and three ABC-DLBCL lines (OCI-LY3 and OCI-LY10 given by Pr. Feuillard, UMR CNRS 7276, Limoges University, with the kind agreement of the Louis M. Staudt, National Cancer Institute, USA- and U2932 from DSMZ) were used. FL3 was synthesized according to an established procedure [30]. Cells were grown in RPMI 1640 medium (Lonza) supplemented with 10% heat-inactivated fetal calf serum (Hyclone), 100 U/ml penicillin, and 100 μg/ml streptomycin (Gibco). Cell lines were expanded upon receipt and low-passage vials were stored in liquid nitrogen. All experiments were realized within 8 weeks after drawing. The cell lines were routinely tested to confirm the absence of mycoplasma by the MycoAlert Kit from Lonza. For cell viability assays, 5 × 10^4^ cells/well were seeded in flat-bottom 96-well microtiter plates (Starsted, Inc.) and FL3 (0–100 nM) or solvent (DMSO) was added in triplicate. Cell viability was assessed after 48 and 72 h using the XTT assay (Cell Proliferation Kit II, XTT Roche) according to the manufacturer’s instructions. Results are expressed as the relative cell viability (ratio) compared to untreated control cells. Nonlinear dose-response curves were fitted to the data using Graphpad Prism Version 5 software. From these curves, GI50 (the concentration of FL3 at which the fraction of affected cells is 50%) and Emax values (the fraction of affected cells at the maximum effect of FL3) were calculated for each DLBCL cell line tested.

### Analysis of apoptosis

DLBCL cells (1 × 10^6^ cells/mL) were treated with FL3 (1–20 nM). After 48 h and 72 h, apoptosis was determined using the propidium iodide (PI)/Annexin V-FITC double staining method (kit Beckman Coulter, Immunotech) as described previously [31].

### Subcellular fractionation

For analyzing intracellular trafficking of AIF and PHB1, subcellular fractionation was performed using a nuclear/cytoplasmic fractionation kit (Thermo Fisher Scientific), according to the manufacturer’s protocol. We used GAPDH or Hsp90 (Sigma Aldrich) and Histone 3 antibodies (Santa Cruz) as controls for the cytoplasmic and nuclear fractions, respectively.

### Western blotting

Proteins were obtained from whole cell lysates using lysis buffer (20 mM Tris–HCl [pH 7.5], 1% NP-40, 10% glycerol, and 150 mM NaCl supplemented with 1% protease and phosphatase inhibitor mixtures) (Sigma) as previously described [28]. The primary Abs used were: mouse anti-Akt1/2, rabbit anti-phospho-Ser473Akt, rabbit anti-Erk1/2 or anti-phospho-Erk1/2, anti-Mnk1, anti-4E-BP1, anti-AIF, anti-PHB1, anti-PHB2, anti-phospho(Ser/Thr)-Akt substrate, anti-eIF4E, anti-Phospho-eIF4E, anti-C-Raf and anti-phospho-C-Raf (Ser289/296/301) (Cell signaling Technology), mouse anti-PARP, rabbit anti-Bcl-2 and anti-c-Myc (Santa Cruz Biotechnology), and anti-βactin (Sigma). Horseradish peroxidase (HRP)–conjugated secondary antibodies (1:1000) were from DakoCytomation. Blotted proteins were detected and quantified using the Immobilon Western Chemiluminescent HRP Substrate (Millipore) and a bioimaging system (Genesnap; Syngene).

### 7-Methyl-GTP cap binding assay

5 × 10^5^ cells were washed in PBS and resuspended in lysis buffer. After centrifugation, (16,000 x g for 10 min at 4 °C) 200 μg protein was applied to 20 μL immobilized γ-aminophenyl-7-methyl-guanosine 5′-triphosphate (m7GTP) agarose beads (Jena Bioscience) and incubated for 3 h at 4 °C. The samples were analyzed for eIF4A and eIF4E by western blotting.

### Real-time quantitative PCR analysis

Total RNA was isolated using the QIAzol Lysis Reagent from QIAGEN, and 2 μg of total RNA was used as template for cDNA synthesis using the High-Capacity cDNA Reverse Transcription Kit (Appleid Biosystems), according to the manufacturer’s instructions. Real-time PCR reactions were conducted in triplicate with TaqMan® Fast Universal PCR Master Mix (Applied Biosystem ThermoFisher Scientific) and *AKT1*, *AKT2*, *MNK1* or 18S TaqMan® Gene Expression Assays (Primer/Probe Set). Amplification of cDNA was measured using a StepOnePlusTM Real-Time PCR System (Applied Biosystem). Quantitation of results was determined using the delta delta CT method. *AKT1, AKT2* and *MNK1* mRNA expression in DLBCL cells was normalized to *18S* RNA levels (delta CT) and then expressed relative to culture condition control (delta delta CT).

### Immunohistochemistry (IHC)

IHC stainings were performed as previously described [32] using the Envision Kit (Dako Denmark A/S). Rabbit anti-PHB1, anti-PHB2, anti-AIF antibodies were purchased from Cell Signaling Technology and mouse anti-Ki67 was obtained from Dakko. Mouse and rabbit isotypic control antibodies were purchased from Sigma. For PHB expression in biopsy samples, the staining intensity was scored from 0 to 4 as follows: 0, negative; 1, weak; 2, moderate; 3, strong; 4, very strong. All tumor samples were PHB1 positive. For statistical analysis, a staining index score ≥ 3 indicated tumors with high PHB1 or PHB2 expression, and < 3 low PHB1 or PHB2 expression.

### In vivo xenografts

All animal studies were conducted in accordance with the guidelines established by the internal Institutional Animal Care and Use Committee (CREEAL N°2-07-2012). Four-week-old SCID female mice (CB17.SCID) were supplied by Janvier Labs. Mice were inoculated with 1 × 10^7^ SUDHL4 cells subcutaneously. After the tumors had become established (~ 6 weeks) i.p. administration of FL3 (15 mg/kg diluted in DMSO/PBS with 5% Cremophor) or vehicle alone (DMSO/PBS with 5% Cremophor) was performed two times per week for 2 weeks. Mice used in the study were monitored every other day. No sign of toxicity in the animals and no unexpected death were observed throughout the experiment. Moreover, no change in body weight of mice was detected (data not shown). The tumor volume (cm^3^) was estimated by three weekly measurements and calculated using the formula length x width x height. Mice were killed after 13 days of treatment and tumor samples were harvested for analysis.

### Analysis of CD20 cell surface expression

Surface expression of CD20 on the ABC cell line U2953 was assessed as compared to SUDHL4 cells by flow cytometry. 10^6^ cells were incubated with anti-CD20-PE specific Ab (Beckman Coulter) or isotypic control Ab (mouse IgG2a-PE, Beckman Coulter) for 30 min at 4 °C. After washing, cells were resuspended in PBS/0.1% PFA and analysed with a FACSCalibur flow cytometer (Becton Dickinson, Heidelberg, Germany) acquiring 10,000 events gated on light scatter properties (forward vs side: FSC/SSC) to eliminate debris and cellular aggregates.

### Statistical analysis

Statistical analyses were performed using StatView v5.0 software (SAS Institute; Cary, NC). Significance between groups in the in vitro and xenograft studies was determined using the Student’s t-test. For the DLBCL patient studies, correlations between quantitative variables were assessed using the Spearman rank correlation coefficient. A Chi square test (χ^2^-value) was used to analyze the association between PHB expression and clinicopathological parameters. The duration of overall survival (OS) was calculated from the date of diagnosis to the date of last follow-up or death. Event-free survival (EFS) was defined as time from diagnosis until relapse or progression or death as a result of any cause or to the date on which the study was stopped. Kaplan-Meier survival curves were used to estimate OS and EFS rates, and the log-rank (Mantel-Cox) test was used to assess differences in survival between groups. *P* values < 5% were considered to be statistically significant.

## Results

### Expression of PHB1 and PHB2 in DLBCL cell lines and tumor cells from patients

**Fig. 1:**
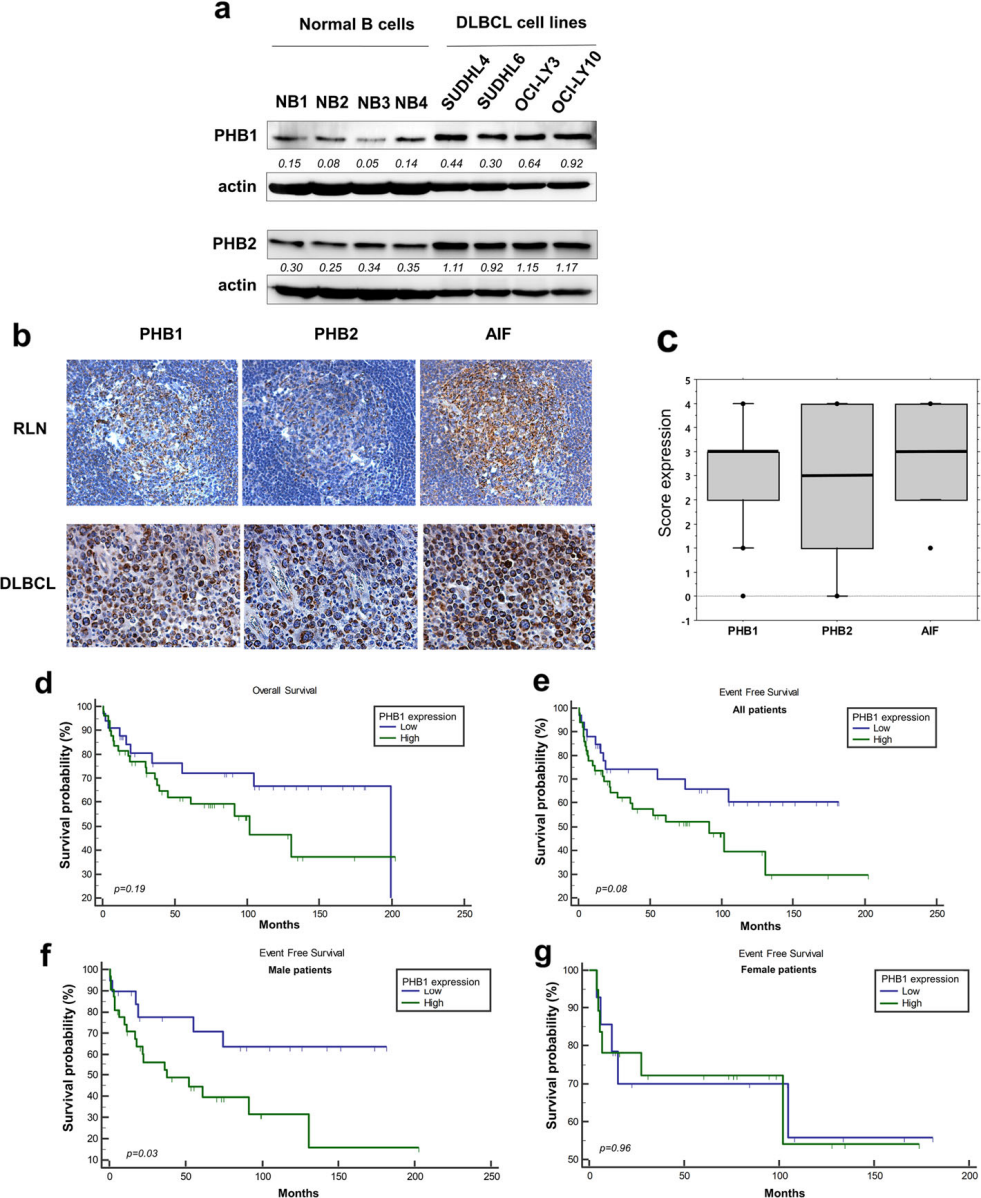
Expression of PHB1 and PHB2 in DLBCL cell lines, normal B cells and patient samples. **a** Expression of PHBs were analyzed by Western blotting in cell lysates of GCB (SUDHL4, SUDHL6) and ABC (OCI-LY3, OCI-LY10) DLBCL cell lines, as compared to peripheral normal B lymphocytes isolated from PBMCs of 4 healthy donors (of whom NB1 and NB2 were female and NB3 and NB4 were male donors). Actin was used as a loading control. The Western blots have been quantified by densitometry and normalized to actin intensity. The quantitative values have been incorporated below the Western blots bands. **b** Examples of immunohistochemical stainings of PHB1, PHB2, and AIF in DLBCL tumor samples (× 200) and normal reactive lymph nodes (RLN, × 200, *n* = 3). **c** Box-plot analysis showing the distribution of total immunostaining scores determined for the tumor samples (*n* = 82). The horizontal line corresponds to the median value and the box length to the interquartile range. Box whiskers represent the maximum and minimum range excluding extreme outliers (shown as dots). Kaplan-Meier plots of OS (**d**) and EFS (**e**) in patients with DLBCL (*n* = 82) stratified by PHB1 expression level, as indicated in the Methods section. Kaplan-Meier plots of EFS in **f** male (*n* = 48) and **g** female patients (*n* = 34)

PHB1 and PHB2 expression was analyzed in GCB (SUDHL4, SUDHL6 cell lines) and ABC (OCI-LY3, OCI-LY10 cell lines) subtypes of DLBCL. SUDHL6 and SUDHL4 cell lines are in vitro models of DHL and THL subgroups respectively [33]. PHB1 and PHB2 were found expressed in all DLBCL cell lines and levels of PHB1 and PHB2 were higher than in normal peripheral B cells (Fig. 1a). As expected, similarly to AIF (Apoptosis inducing factor) (Additional file 1: Figure S1), PHB proteins are primarily confined to the mitochondria in all cell lines tested, as revealed by the colocalization of PHB1 staining with the mitochondrial marker (Tom20). We further assessed PHB1 and PHB2 expression in 82 patient samples, comprising 49 belonging to the GCB subtype and 33 to non-GCB subtype (Table 1). Normal reactive lymph nodes (RLN) were taken as control samples. As expected, we observed some positive stainings for PHB1 and, to a lesser extent, PHB2 in most B cells within the germinal centers of RLNs (Fig. 1b). Although variable, moderate to high IHC intensity was observed for PHB1 and PHB2 in DLBCL cells (mean score of 2.7 ± 0.1 and 2.3 ± 0.1 for PHB1 and PHB2 respectively, Fig. 1c). We mostly detected a strong immunoreactivity in perinuclear mitochondria similar to the expression of the mitochondrial protein AIF (Fig. 1b). PHB1 and PHB2 staining strongly correlated with each other and with AIF staining (Table 2). Of interest, PHB2 expression was significantly correlated with age-adjusted International Prognostic Index and elevated serum LDH (LDH ratio to the upper limit of normal ULN) (Table 2). PHB1 staining score was correlated with elevated serum LDH (Table 2) and patients with high serum LDH usually presented higher PHB1 stainings (Additional file 1: Table S1). Finally, even though Ki-67 scores were not available for all patients, high Ki-67 expression was associated with high PHB2 stainings (Additional file 1: Table S2). No differential PHB expression was identified in relation to DLBCL subtype or other clinic pathological features (Additional file 1: Table S1). Kaplan-Meier survival analysis showed that high PHB1 expression tended to be associated with shorter EFS but not OS (Fig. 1d and e). However, when female and male patients were analyzed separately, high PHB1 predicted poorer EFS in men but not in women patients (Fig. 1f and g). Collectively, these results strongly suggest that high level of PHBs may be involved in tumorigenesis and the clinical course of DLBCL.

**Table 2:** Correlation between immunohistochemical scores and clinical DLBCL patient characteristics

	PHB2	AIF	IPI	IPIaa	LDH^a^
PHB1					
Correlation coefficient	0.471	0.338	0.148	0.135	0.264
*p* value	0.00001	0.002	0.183	0.224	0.016
PHB2					
Correlation coefficient		0.561	0.203	0.257	0.313
*p* value		0.00000004	0.066	0.019	0.004

Spearman’s rho coefficient and *p*-value as determined by the Spearman rank correlation test

^a^LDH: ratio to ULN (Upper limit of the normal)

### The PHB ligand, FL3, induced apoptosis in DLBCL cell lines associated with nuclear translocation of PHB1 and AIF

**Fig. 2:**
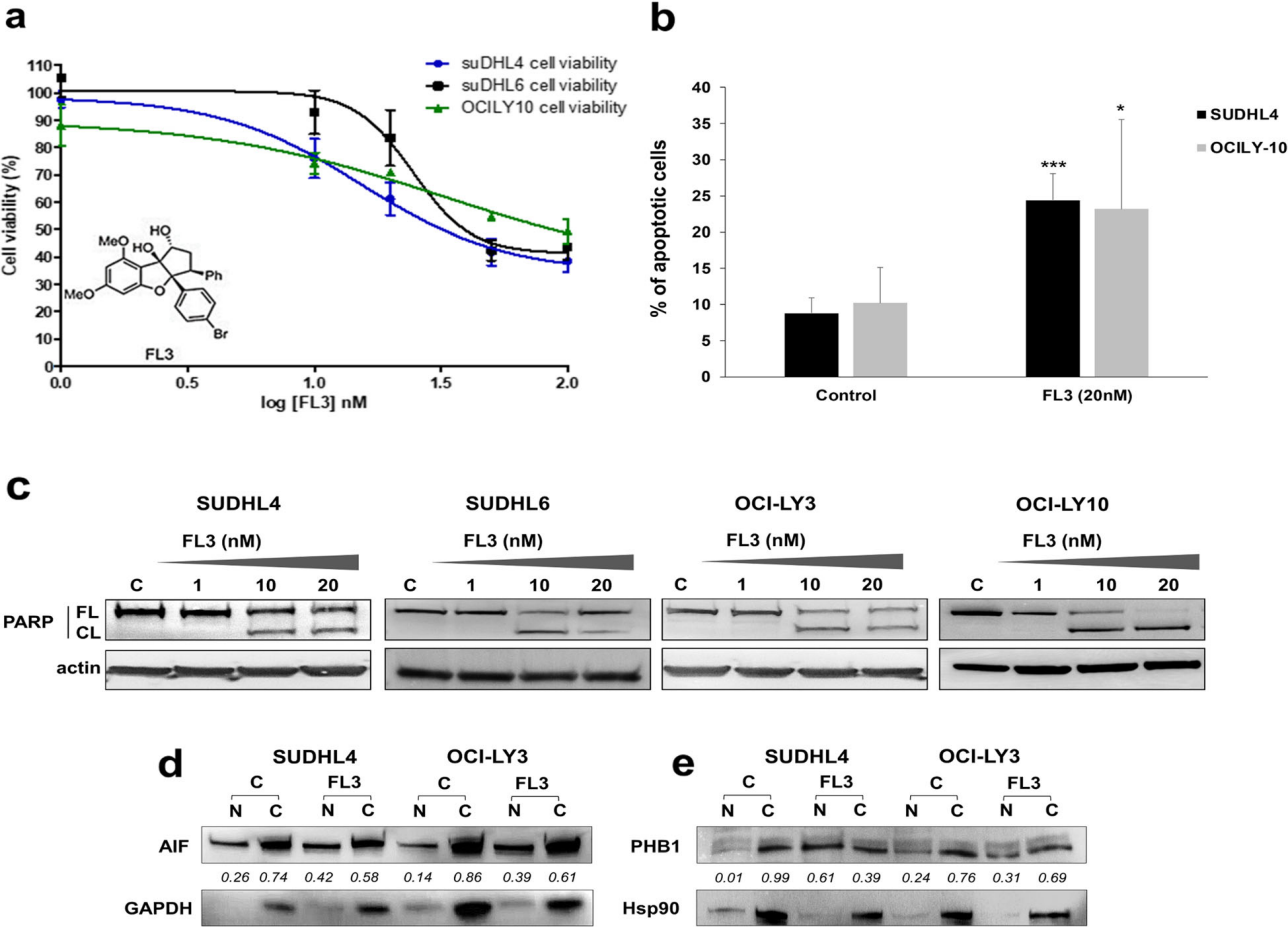
The PHB ligand, FL3, induces apoptosis in GCB and ABC-DLBCL cell lines that is associated with nuclear translocation of AIF and PHB1. **a** Dose-response of GCB (SUDHL4/6) and ABC (OCI-LY10) DLBCL cell-line viability after 72 h FL3 treatment (1–10–20-50-100 nM) using the XTT test. The plot shows the means ± SEM of four independent experiments performed in triplicate. Inset: The chemical structure of FL3. **b** Flow cytometry analysis of apoptosis in cell cultures exposed or not (DMSO control) to 20 nM FL3 for 72 h by PI/Annexin V-FITC double staining. Results are shown for the SUDHL4 and OCI-LY10 cell lines as the mean percentages of apoptotic cells of five independent experiments. *, *** indicate significant differences compared to control with *p* < 0.05 and *p* < 0.001 respectively. **c** Apoptosis was also confirmed for all DLBCL cell lines by Western blot analysis of poly (ADP-ribose) polymerase (PARP) cleavage in cell lysates as shown from 72 h non-treated or FL3 treated cells (1–20 nM). Data are representative of three independent experiments. FL: full-length (FL), CL: cleaved PARP. Actin was used as a loading control. The intracellular localization of **d** AIF and **e** PHB1 was studied in SUDHL4 and OCI-LY3 cell lysate nuclear (N) and cytosol (C) fractions after 72 h of FL3 exposure by Western blot analysis. GAPDH and Hsp90 expressions were used to check the purity of fractions. For each condition, relative densitometric quantification was realized and the ratio to total PHB1 or AIF expression was calculated. Results are representative of two independent experiments

We evaluated the anti-tumor effect of FL3, a synthetic flavagline and ligand of PHBs that was shown to interfere with membrane localization of PHB [17, 30, 34]. Cytotoxicity of FL3 (1–100 nM) was evaluated using the XTT assay and Annexin V-FITC/PI dual staining on GCB (SUDHL4 and SUDHL6) and ABC (OCI-LY3 and OCI-LY10) cell lines. Since OCI-LY3 cells form big clumps in cultures, evaluation of cell apoptosis was analyzed by western blotting to monitor the cleavage of the caspase substrate polyADP-ribose polymerase (PARP). FL3 significantly reduced cell viability with 50% growth inhibition (GI_50_) values between 15 and 33 nM after 72 h of treatment (Fig. 2a, Table 3). Cytotoxicity slightly increased in a dose- and time-dependent manner, but cell lines were relatively resistant to high concentrations of FL3 with a maximum of 51 to 61% cells affected (E_max_) at 100 nM (Table 3). We further investigated the cell signaling pathways involved in FL3-mediated cytotoxicity. FL3 induced apoptosis in all tested cell lines as shown in Fig. 2b for SUDHL4 and OCI-LY10 with the dose of 20 nM. This effect was confirmed in GCB and ABC cell lines by detection of PARP cleavage (Fig. 2c). Translocation of PHB1 and the mitochondrial protein AIF between mitochondria and the nucleus, or from cytoplasm to nucleus, was proposed to be part of the apoptotic signaling in normal and cancer cells, notably following flavagline exposure [19, 30, 34]. We thus analyzed the subcellular localization of PHB1 and AIF in SUDHL4 and OCI-LY3 cells upon exposure to FL3. After treatment with FL3, a fraction of AIF and PHB1 translocated to the nucleus (Fig. 2d and e). This effect was also confirmed in SUDHL6 cells (Additional file 1: Figure S2). Moreover, confocal analysis showed that PHB1 peak staining was no longer colocalized with that of the mitochondrial marker (Tom20) when cells were exposed to FL3 (data not shown). Of note, 24 h and 72 h FL3 exposure did not influence total PHB1 and PHB2 cell content (Additional file 1: Figure S3). These data show that FL3 determines PHB1 relocalization outside mitochondria in DLBCL cells; furthermore, they suggest that PHB1 and AIF translocation to the nucleus may be involved in FL3-induced apoptosis of DLBCL cells.

**Table 3:** Cytotoxicity of FL3 in DLBCL cell lines

	FL3 GI_50_	FL3 E_max_
SUDHL4	15.39 nM	61%
SUDHL6	24.53 nM	56%
OCI-LY10	32.52 nM	51%

The table shows 50% growth-inhibition values (GI_50_) and maximum affected cells (Emax) for each DLBCL cell line

### FL3 inhibits ERK–MNK–eIF4E signaling pathway in DLBCL cell lines

**Fig. 3:**
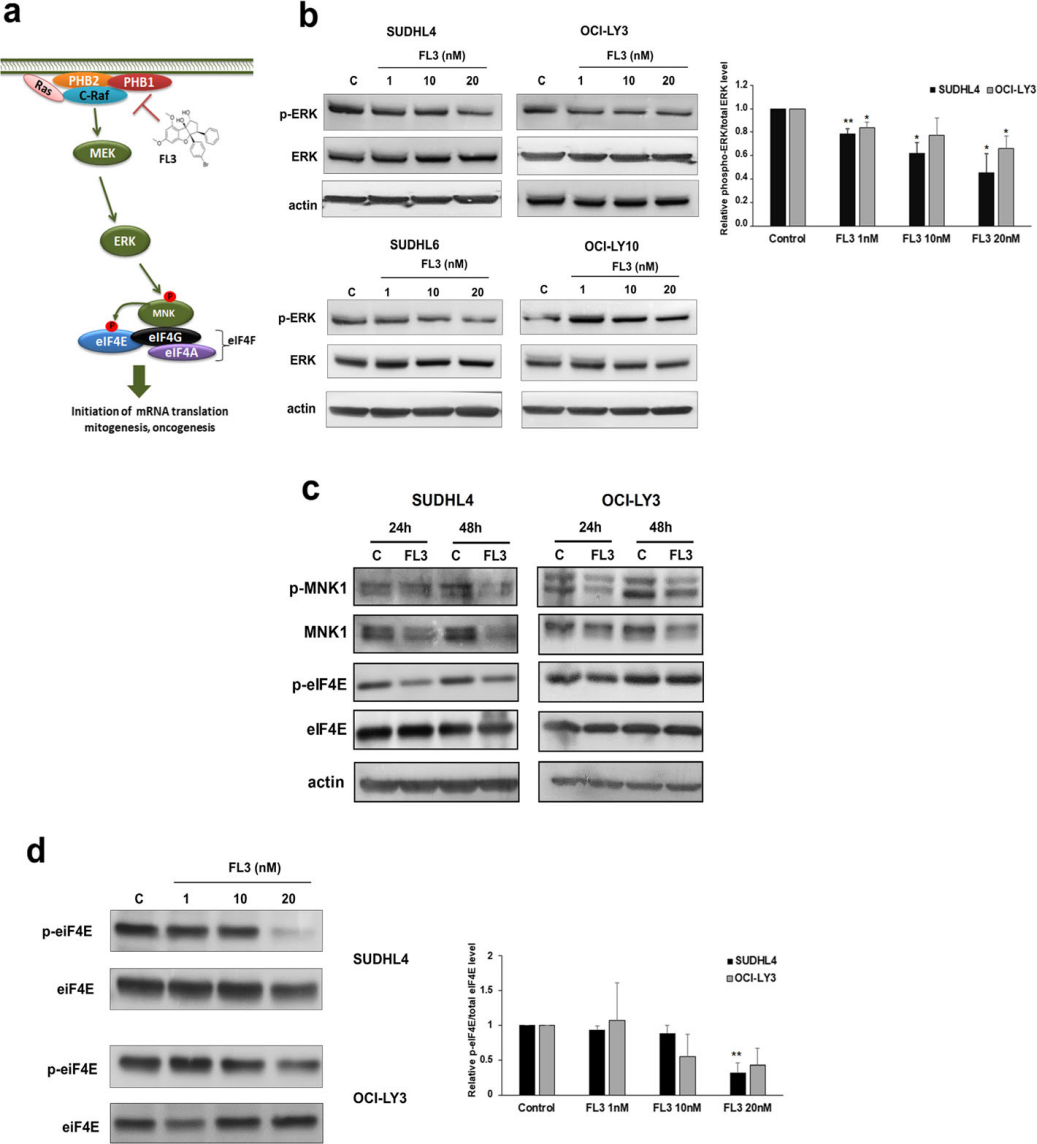
FL3 reduces ERK–MNK–eIF4E signaling pathway in DLBCL cell lines. **a** Schematic representation of the FL3 mechanism in disrupting PHB-C-Raf interaction and thus in C-Raf-MEK-ERK signaling pathway. **b** ERK1/2 (ERK) and phospho-ERK1/2 (p-ERK) expression determined by Western blotting after 72 h treatment (FL3, 1–20 nM) as compared to control (C, DMSO control) in GCB (SUDHL4, SUDHL6) and ABC (OCI-LY3, OCI-LY10) cell lysates. Densitometry analysis of phospho-ERK to total ERK levels is shown for SUDHL4 and OCI-LY3 and data were expressed as the means ± SD of three independent experiments. Actin was used as a loading control. *, ** indicate significant differences to control with *p* < 0.05 and *p* < 0.01 respectively. **c** Immunoblot analysis of phospho-MNK1, MNK1, phopho-eIF4E and eIF4E protein levels in SUDHL4 and OCI-LY3 cell lysates representative of 3 independent experiments. **d** SUDHL4 and OCI-LY3 cell lines were cultured with or not (C, DMSO control) FL3 (1–20 nM). Representative immunoblots of total and phospho-eIF4E protein levels of three independent experiments are shown. Histograms show quantification by densitometry of phospho-eIF4E normalized to total eIF4E levels. Data are the means ± SD. ** *p* < 0.01 compared to control

PHBs are adaptor proteins in membrane-associated signaling pathways involved in maintaining cellular survival [22], in particular by allowing C-Raf activation that can be disrupted by FL3 binding (Fig. 3a). We confirmed in GCB and ABC cell lines that FL3 treatment reduced ERK1/2 phosphorylation in a concentration-dependent manner by western blot (Fig. 3b). Moreover, C-Raf phosphorylation was reduced in cell lysates exposed to FL3 in comparison to controls (Additional file 1: Figure S4). These data strongly suggest involvement of PHB-dependent regulation of the c-Raf-MEK-ERK-MNK1 pathway, and thus phosphorylation of eukaryotic initiation factor 4E (eIF4E). As shown in Fig. 3c, a reduced level of MNK1 phosphorylation was also observed after FL3 treatment, that was associated with a strong reduction in MNK1 protein level in the GCB cell lines and to a lesser extent in the ABC cell line OCI-LY3 (Fig. 3c and Additional file 1: Figure S5). Of note, in SUDHL4 cell line, mRNA level of *MNK1* was significantly decreased following 20 nM FL3 exposure (Additional file 1: Figure S5). Finally, FL3 treatment strongly reduced eIF4E phosphorylation protein level in SUDHL4 cells and, to a lesser extent, in OCI-LY3 cells*.* This effect was detected from 24 h (Fig. 3c) and at 72 h FL3 exposure (Fig. 3d, with *p < 0.01* and *p = 0.05* at 20 nM FL3 for SUDHL4 and OCI-LY3 respectively).

### eIF4F complex formation and activity are altered in DLBCL cell lines treated with FL3

**Fig. 4:**
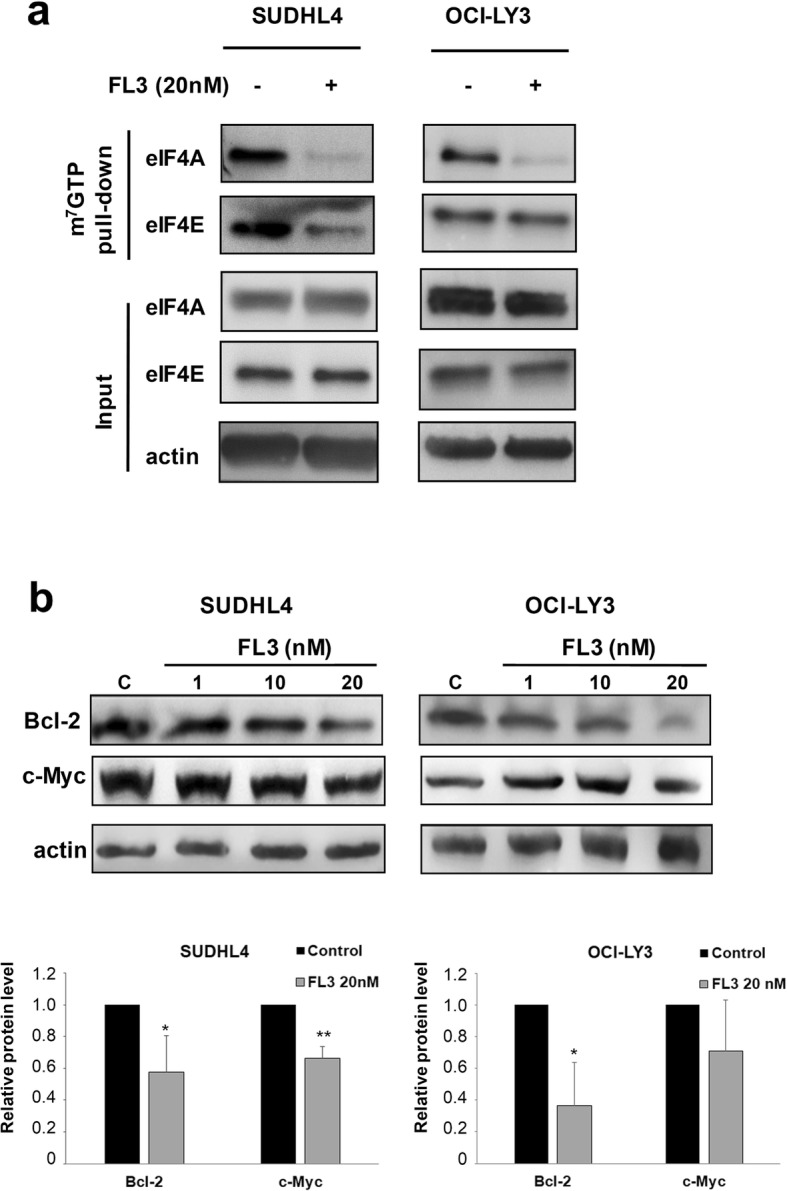
FL3 disrupts eIF4F complex and inhibits cap-dependent translation in DLBCL cell lines. **a** SUDHL4 and OCI-LY3 cells were cultured with or without FL3 (20 nM). Protein lysates from cell cultures were harvested following 72 h treatment and incubated with m7GTP agarose beads overnight at 4 °C. Western blot analysis was performed to observe eIF4A and eIF4E binding to m7GTP in eluates from beads (m7GTP pull-down) and total extracts (input). Data are representative of two independent experiments. **b** Immunoblot analysis of Bcl-2 and c-Myc protein levels in SUDHL4 and OCI-LY3 cell cultures in the presence or not to FL3 (1–20 nM). Actin was used as a loading control. Densitometric analysis of Bcl-2 and c-Myc levels normalized to actin is shown relative to control. Results are presented as means ± SD of 4 independent experiments. *p < 0.05, **p < 0.01 vs control

As eIF4E phosphorylation is involved in the eIF4F complex activity, and because FL3 was also demonstrated to inhibit translation initiation by binding to eIF4A [35], we examined whether the formation of eIF4F complex was involved in the cell sensitivity of DLBCL to FL3. We determined the status of the eIF4F complex in SUDHL4 and OCI-LY3 cells using synthetic m7GTP-agarose beads to capture eIF4E and its binding partners (i.e. eIF4A). FL3 depleted eIF4A from the eIF4F complex in both cell lines tested, and reduced eIF4E cap-binding activity notably in SUDHL4 (Fig. 4a). Accordingly, we observed a reduction in the protein expression of Bcl-2 and c-Myc following FL3 exposure in SUDHL4 cells confirming that FL3 alters cap-dependent translation in DLBCL cells (Fig. 4b; OCI-LY3 cells displayed reduced Bcl-2 levels only).

### Akt protein level is strongly down-regulated after FL3 treatment in DLBCL cell lines

**Fig. 5:**
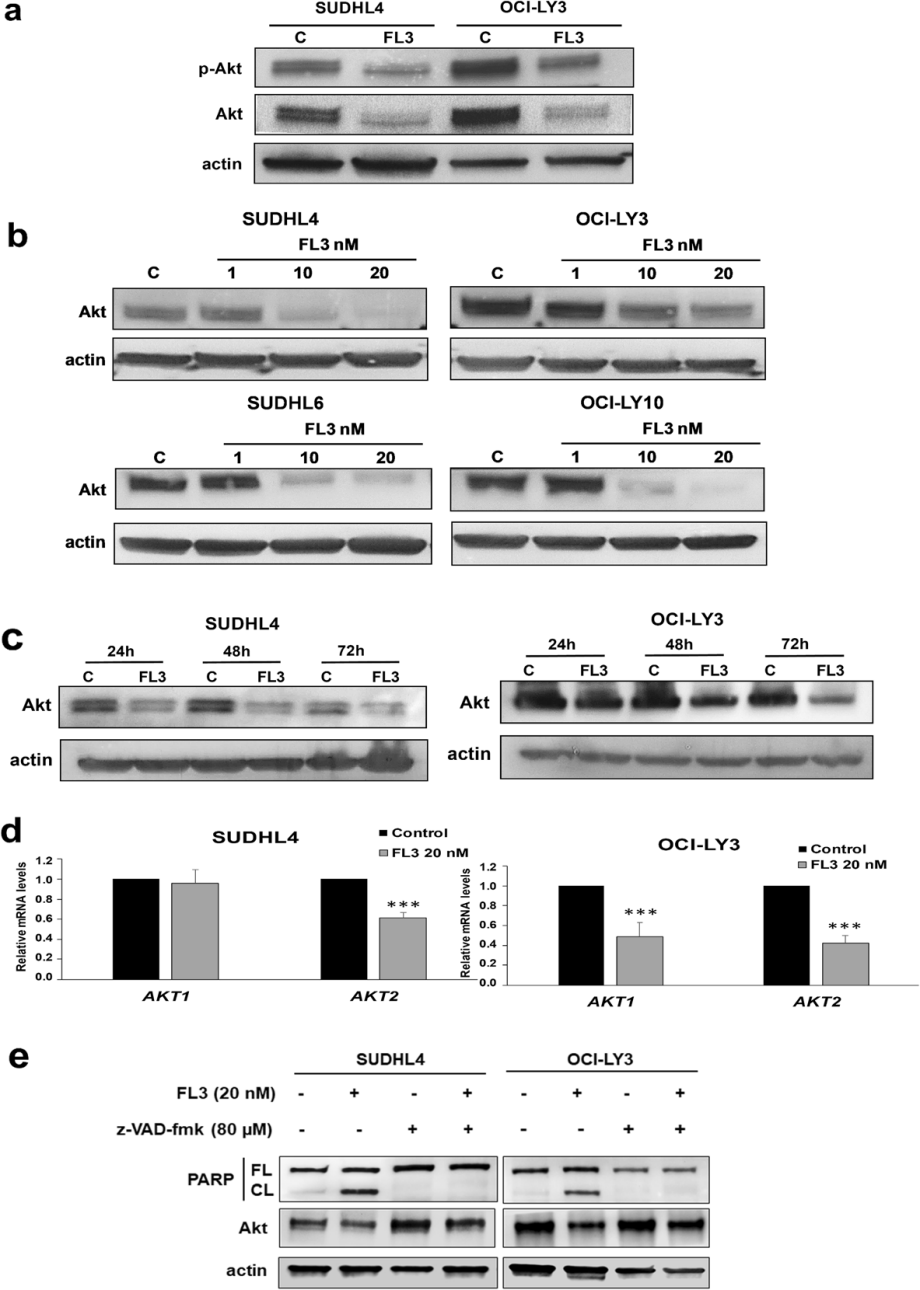
FL3 decreases Akt protein level in DLBCL cell lines. **a** Cell lysates from FL3-treated SUDHL4 and OCI-LY3 cell lines were subjected to Western blot analysis of total (Akt) and phospho-Ser473-Akt (p-Akt) protein level**.** Actin is shown as a loading control. Results are representative of three independent experiments. **b** A concentration-dependent effect of FL3 on Akt protein level was observed in all GCB and ABC DLBCL cell lysates. **c** SUDHL4 and OCI-LY-3 cells were treated for 24, 48 and 72 h with or without 20 nM of FL3 and Akt protein levels were analyzed by Western blot. **d** Relative quantification by qRT-PCR analysis of *AKT1* and *AKT2* mRNA in SUDHL4 and OCI-LY3 cell cultures in presence or not of FL3 (20 nM). Results are expressed relative to control cultures as the means ± SD of six independent experiments. ***: *p* < 0.001 respectively vs control. **e** The decrease in Akt protein level was partially prevented by 2 h pre-treatment of SUDHL4 and OCI-LY3 cells with the pan-caspase inhibitor, z-VAD-fmk (80 μM), before 24 h exposition with FL3 (20 nM). Apoptosis was evaluated by Western blot analysis of PARP cleavage in cell lysates. Data are representative of two independent experiments. FL: full-length (FL), CL: cleaved PARP. Actin was used as a loading control

Constitutive activation of PI3K/Akt pathway is an important oncogenic signaling pathway in both GCB and ABC subtypes of DLBCL [6]. As for ERK1/2, we confirmed the presence of activated p-Akt in DLBCL control cell lysates of GCB and ABC subtypes (Fig. 5a). Interestingly, a large reduction of Akt phosphorylation was observed after FL3 exposure. However, in contrast to ERK1/2, this effect was associated with a strong reduction of Akt protein levels in both GCB and ABC cell lysates relative to controls (Fig. 5a). This effect was more pronounced for Akt than for MNK1 and did not affect other proteins, such as ERK1/2, actin (Figs. 3 and 4), GAPDH, or PI3K (data not shown). The decrease in Akt level was FL3 dose-dependent (Fig. 5b) in all tested cell lines and, as for MNK1, was detected from 24 h FL3 exposure (Fig. 5c). Of note, mTOR phosphorylation (Ser2448) was not affected by FL3 treatment (data not shown). Reduction of Akt protein level was associated with a significant decrease in mRNA level of *AKT2* in SUDHL4 and both *AKT1* and *AKT2* mRNA levels in OCI-LY3 (Fig. 5d). Furthermore, pretreatment of DLBCL cells with 80 μM of z-VAD-fmk, a pan-caspase inhibitor, abrogated FL3-induced apoptosis and prevented partially the reduction of Akt cell level (Fig. 5e). These results indicate that down regulation of Akt1/2 level in DLBCL cells exposed to FL3 may be mediated at least by reduction of *AKT* mRNA levels and by a caspase-dependent cleavage of the protein. Finally, as PHBs are known to be substrates of Akt [36, 37], we indirectly analyzed PHB1 phosphorylation and our results strongly suggest decreased PHB phosphorylation in FL3-treated DLBCL cells (Additional file 1: Figure S6).

### FL3 activity is maintained in cell line resistant to rituximab

**Fig. 6:**
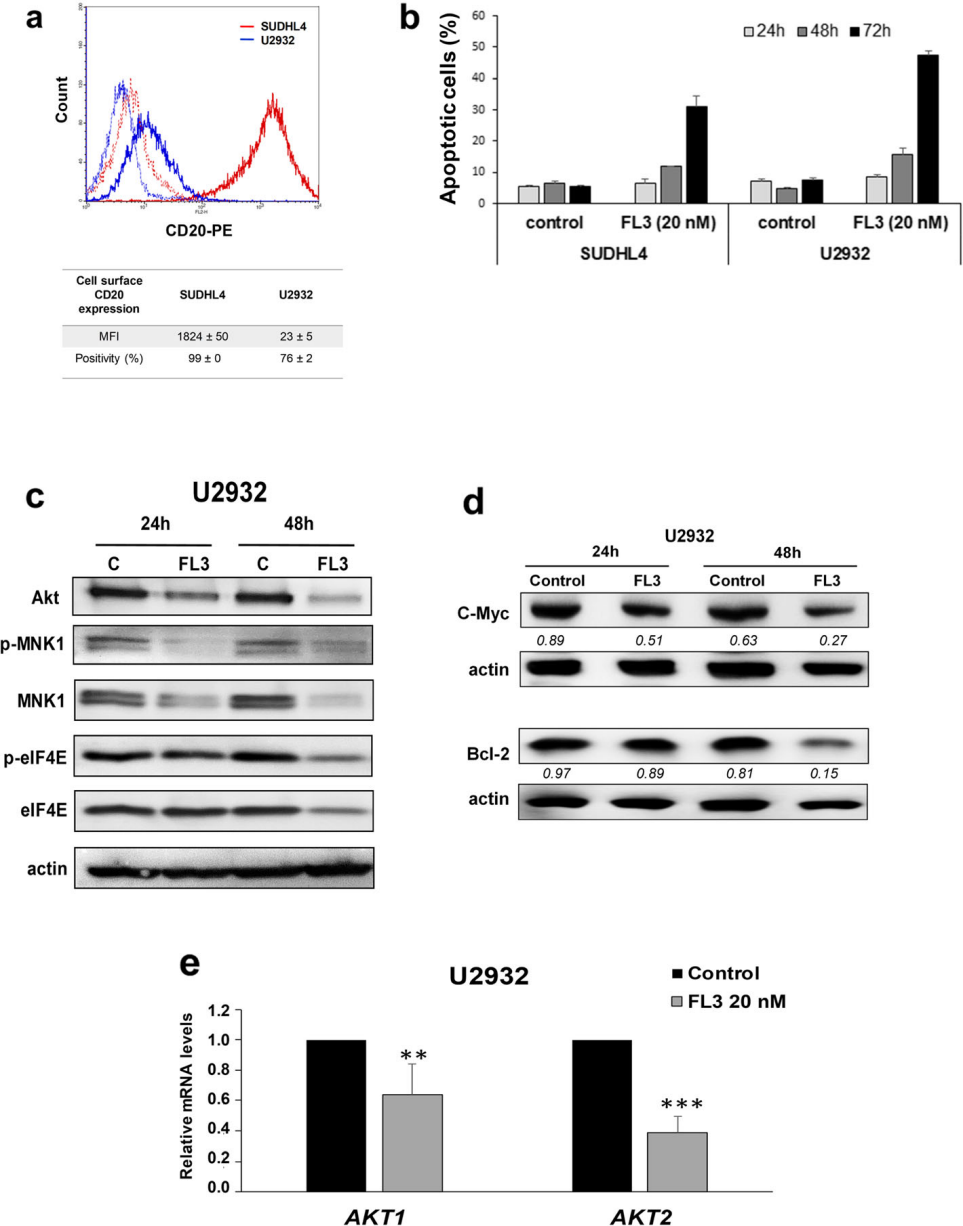
Effects of FL3 in a rituximab resistant cell line. **a** CD20 surface expression was determined using anti-CD20-PE by flow cytometry in U2932 cells as compared to the SUDHL4 cells. Representative histograms are shown: solid outline histograms indicate expression of CD20 in SUDHL4 cells (red) and U2932 (blue); dashed lines are the fluorescence intensity of SUDHL4 (red) and U2932 (blue) cells labeled with an isotype control mAb. Means ± SD of MFI (mean fluorescence intensity) and percentages of CD20+ cells are also done (*n* = 3). **b** The apoptotic response induced by 20 nM of FL3 was analyzed in SUDHL4 and U2932 cells at 24, 48 and 72 h by flow cytometry using the PI/Annexin V-FITC double staining as in Fig. 2. The values represent means ± SD of three independent experiments. **c** Akt, phopho-MNK1, MNK1, phospho-eIF4E, and eIF4E protein levels were analyzed by Western blotting after 24 and 48 h treatment (FL3, 20 nM) as compared to control (C, DMSO control). **d** Immunoblot analysis of Bcl-2 and c-Myc protein levels in U2932 cell cultures after 24 h and 48 h of exposure or not to FL3 (20 nM). Actin was used as a loading control. Densitometric analysis of Bcl-2 and c-Myc levels normalized to actin is indicated below the Western blot bands. **e** Relative quantification by qRT-PCR analysis of *AKT1* and *AKT2* mRNA in U2932 cell cultures in presence or not of FL3 (20 nM). Results are expressed relative to control cultures as the means ± SD of six independent experiments. **, ***: *p* < 0.01 or *p* < 0.001 respectively vs control

Loss of CD20 on the cell surface of tumor cells is one of the major mechanisms of rituximab resistance in DLBCL [38]. In U2932 ABC cell line, that expressed very low level of CD20 (Fig. 6a), FL3 induced a strong apoptotic response (Fig. 6b) compared to SUDHL4 and rituximab (Additional file 1: Figure S7). As for all DLBCL tested, this effect was associated with a reduction of phospho-C-Raf (Additional file 1: Figure S4), phospho-PHB1 (Additional file 1: Figure S6), Akt, MNK1, phospho-eIF4E, Bcl-2 and c-Myc protein expression (Fig. 6c and d), and a significant decrease in *AKT1* and *AKT2* mRNA levels (Fig. 6e).

### FL3 reduces tumor growth in a DLBCL xenograft model

**Fig. 7:**
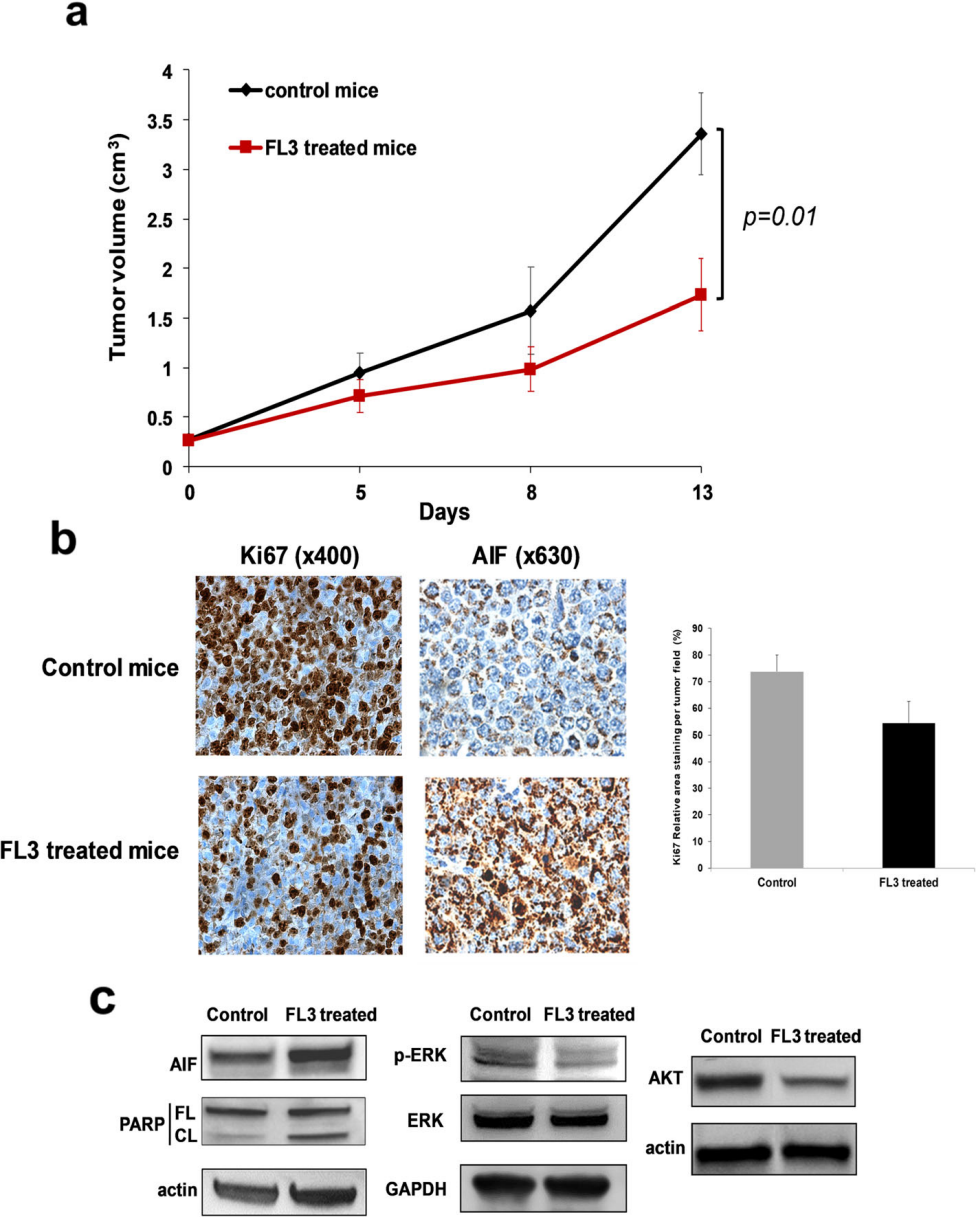
FL3 inhibits the growth of DLBCL tumors xenografted in mice. SCID mice bearing tumors from SUDHL4 cells were injected i.p. with either vehicle or FL3 (15 mg/kg) twice per week for 2 weeks. **a** The means ± SEM of tumor volume from the mice are shown. **b** Tumors were immunostained for Ki67 and AIF. IHC photographs are representative fields from control and FL3 treated animals. Images of IHC staining of Ki67 was quantified using ImageJ software and data are expressed as means ± SD of 3 independent fields of each tumor sample for the 2 control and the 2 FL3 treated mice. **c** Western blotting was performed to detect the protein expression levels of the indicated molecules from tumor samples. Actin or GAPDH was used as a loading controls. Representative data of two independent experiments are shown for vehicle control- and FL3-treated mice

Finally, we used the SUDHL4 xenograft model in SCID mice to investigate the efficacy of FL3 in vivo. Treatment with FL3 (15 mg/kg, i.p.) induced a significant inhibition of DLBCL tumor growth (Fig. 7a) (1.64 vs 3.36 cm^3^ at day 13). This effect was associated with reduced Ki-67 expression (Fig. 7b) and increased PARP cleavage (Fig. 7c) demonstrating apoptosis in tumor samples of FL3 treated mice. We also observed strong localization of AIF in the nucleus of several tumor cells from FL3 treated mice by IHC analysis (Fig. 7b). Ex vivo analysis confirmed that FL3 reduced phosphorylation of ERK1/2 and decreased Akt expression in tumor samples (Fig. 7c).

## Discussion

The present study was designed to evaluate the clinical significance of PHB expression and the potential therapeutic relevance of targeting PHB in DLBCL. We focused on the flavagline FL3 because of its abilities to bind to PHBs and modulate their functions, and its in vivo antitumor activity [30, 35]. PHB1 and PHB2 protein levels were higher in GCB and ABC cell lines compared to normal peripheral B cells suggesting that PHB1 and PHB2 are upregulated during tumorigenesis. Consistent with this, PHB1 and PHB2 were recently shown to be overexpressed in lymphoid and myeloid tumor cell lines compared to normal naive and activated primary human PBMCs [24]. The PHB protein complex promotes mitochondria stabilization and cell survival in these hematologic malignancies. In the present study, data in sample patients strongly suggest that PHBs are mainly localized in mitochondria with AIF, consistent with what we observed in DLBCL cell lines. We found no significant differences between DLBCL subgroups (GCB vs. non-GCB). However, high expression of PHB1 tended to correlate with an aggressive clinical course. Interestingly, when female and male patients were analyzed separately, high PHB1 expression was a bad prognostic indicator for EFS in men but not in women. This result is not due to sex differences in PHB1 and PHB2 expression in patients (Additional file 1: Table S1), as it was also suggested by data on normal peripheral B cells and DLBCL cell lines (Fig. 1a, where SUDHL4, SUDHL6 and OCI-LY3 are derived from males and OCI-LY10 is derived from female patient). Even though the mechanism remains to be determined, these findings could be explained by the discovery of PHBs in mediating sex dimorphic effects, and notably in immune function [39]. While evidence of a role for PHB1 overexpression has been reported in cancer progression [23, 40, 41], higher levels of PHB2 was found in proliferating cells, including neoplastic tissues [21]. Consistent with this, we show here for the first time in DLBCL that high PHB2 expression is associated with high level of Ki-67; moreover PHB1 and PHB2 score expressions were correlated with marker of tumor burden in DLBCL patients (i.e. elevated serum LDH) and PHB2 expression with IPIaa that remains an important prognostic tool for evaluating DLBCL patients. Collectively, these data strongly suggest that PHB proteins may be key factors in DLBCL cell survival and proliferation.

Mitochondrial localization of PHBs was previously shown to be indispensable for mitochondria integrity and function, and thus cell survival, included in normal B lymphocytes and primary leukemia and lymphoma cells [24, 25]. However, in the cytosol and at the inner face of membrane PHB can interact with several key partner proteins involved in cancer progression. PHBs are notably required for the membrane localization and activation of C-Raf by the oncogene Ras, and consequently for the activation of C-Raf-MEK-ERK signaling [22] that is up-regulated in several cancers, including lymphomas [42]. In this study we used the flavagline FL3 to gain insight into the cytoplasmic role of PHBs in DLBCL cells. Indeed, by binding to PHB, flavaglines were shown to inhibit PHB-mediated signaling pathways, and this interaction did not target mitochondria [36, 43]. As expected, FL3 triggered apoptosis of DLBCL cell lines and also in vivo in a xenograft murine model. Mechanisms involved AIF, as previously reported [30], and a caspase-dependent pathway demonstrated by PARP cleavage. Of interest, aggressive cell lines as ABC-DLBCL cells or representative models of *MYC/BCL-2* DHL and *MYC/BCL-2/BCL6* THL DLBCL subtypes were also sensitive to the FL3-induced apoptotic effect. Importantly, flavaglines included FL3 were shown to inhibit the proliferation of tumor cells without any significant toxicity in vivo [44] and on normal cells, such as human peripheral T, B and bone marrow stem cells [14, 16]. Therefore, our data indicate that targeting PHBs may be a potential therapeutic option for DLBCL notably in aggressive subtypes.

Binding of flavaglines to PHB proteins has been implicated in the inhibition of Raf-MEK-ERK-MNK1-mediated phosphorylation of eIF4E [16, 17, 22]. Our data confirmed reduced activation of c-Raf-ERK1/2-MNK1 pathway in in DLBCL cell lines and for ERK1/2 in the in vivo model. Furthermore, FL3 strongly inhibited eIF4E phosphorylation in GCB cells, which has been implicated in tumorigenesis, including lymphomagenesis. Indeed, eIF4E phosphorylation by MNK1/2 and p38 MAPKs is required for its oncogenic role by translation of a subset of mRNAs encoding proteins involved in survival and tumor invasion [13, 45]. Surprisingly, we found a decrease of total MNK1 level that was associated with a reduced phosphorylation after FL3 exposure. A marked effect was observed on GCB cell lines, which could be explained by the distinct distribution of MNK1 and MNK2 in GCB and ABC DLBCL subtypes respectively [46]. Interestingly, flavaglines have been reported to exert their antitumor activity, including in leukemia and lymphoma mouse models, by binding also to the RNA helicase eIF4A. However this effect may vary between tumor cells. Binding to eIF4A leads to translational inhibition through stabilization of RNA-bound eIF4A in the eIF4F complex, preventing new incorporation of free eIF4A [47–50]. In DLBCL cell lines, FL3 altered eIF4F complex formation and activity. This was confirmed by reduced expression of Bcl-2 and to a lesser extends c-Myc that are involved in the aggressive clinical course and poor prognosis of patients with DLBCL [51]. In ABC cell lines, c-Myc level was differentially influenced by FL3, which could be related to the constitutive activation of the NF-κB pathway in ABC rather than GCB cell lines [52]. The natural flavagline silvestrol, was previously reported to reduce eIF4A helicase translation activity with a marked reduction of c-Myc and Bcl-2 expression in T-ALL and BCR-activated splenic B cells [48, 53]. Our data strongly suggest that FL3 decreases cap-dependent translation in DLBCL cells by binding both to PHBs and eIF4A, which consequently sensitized cells to programmed cell death. However, further studies would be needed to precise their relative contribution which could vary according the DLBCL subtype. Interestingly, phosphorylation of PHBs by Akt was recently demonstrated to be required for PHB translocation and localization in the mitochondria, as well as for tumor cell proliferation [36, 37]. It was proposed that, by inhibiting Akt/PHBs interaction, FL3 may decrease the PHB localization within mitochondria leading to mitochondria dysfunction and thus apoptosis. Indeed, we observed by confocal microscopy analysis, delocalization of PHB1 from mitochondria in cells exposed to FL3 (data not shown). This effect may be emphasized in DLBCL cells by the global reduction level of Akt induced by FL3, that we demonstrate for the first time in the present study. Consistent with this, indirect analysis of PHB1 phosphorylation strongly argues for a FL3-induced reduction of PHB phosphorylation, and cytoplasmic content of PHB1 was decreased following FL3 exposure with a translocation in part to the nucleus. Interestingly, no effect of FL3 (as for rituximab exposure, Additional file 1: Figure S3b) was observed on PHB1 and PHB2 total cellular levels. Nuclear re-localization of PHBs has been associated to their interaction with transcription factors, such as p53 and Rb, promoting apoptosis and inhibition of transcription [19, 54]. This could explain the down regulation of the *AKT1/2* mRNA that we observed in the present work. However, we can’t exclude an apoptosis degradation of *AKT* mRNA [55] as we showed for the AKT protein cell levels. Finally, reduced Akt, Mnk1, Bcl-2 and c-Myc protein expression in association with FL3-induced apoptosis was confirmed in a cell line (U2932) characterized by lower surface CD20 which open the possibility to treat rituximab-resistant diseases.

## Conclusions

This study revealed that PHB overexpression is associated with clinical and biological markers of tumor aggressiveness in DLBCL, especially in male patients. Furthermore, the PHB-ligand FL3 determined anti-tumor activities in vitro and in vivo that were associated with inhibition of ERK/MNK/eIF4E signaling pathways and AKT expression. These effects were observed also in cell lines representative of aggressive DLBCL subtypes. In conclusion, we propose that targeting PHBs may be a promising tool in DLBCL therapeutic strategies particularly for male patients and patients who do not respond to conventional treatments.

## Supplementary information

**Additional file 1.** Supplementary materials.

## Data Availability

The datasets used and analyzed during the current study are available from the corresponding author on reasonable request.

## Abbreviations

ABC: Activated B-cell–like
AIF: Apoptosis activating factor
Bcl2: B cell lymphoma 2
CARD: Caspase Recruiting Domain
CBM: CARD11-BCL10-MALT1
CHOP: Cyclophosphamide, doxorubicin, vincristine, prednisone
DHL: Double-hit lymphoma
DLBCL: Diffuse large B-cell lymphoma
DMSO: Dimethyl sulfoxide
EFS: Event-free survival
eIF4A/E/G: Eukaryotic translation initiation factor 4 A
ERK: Extracellular signal–regulated kinase
FITC: Fluorescein isothiocyanate
GAPDH: Glyceraldehyde 3-phosphate dehydrogenase
GCB: Germinal center B-cell–like
HRP: Horse radish peroxidase
IHC: Immunohistochemistry
IPIaa: Age-adjusted International Prognostic Index
LDH: Lactate dehydrogenase
m7GTP: 7-methyl-guanosine 5′-triphosphate
MNK1: MAPK-interacting Ser/Thr kinase 1
mTor: Mammalian target of rapamycine
NF-κB: Nuclear factor kappa-binding
OS: Overall survival
PARP: polyADP-ribose polymerase
PBMC: Peripheral blood mononuclear cells
PBS: Phosphate-buffered saline
PE: Phycoerythrin
PHBs: Prohibitin
PI: Propidium iodide
PI3K: Phosphoinositol-3-kinase
PTEN: Phosphatase and tensin homolog
qRT-PCR: Quantitative reverse-transcription polymerase chain reaction
R-CHOP: Rituximab, Cyclophosphamide, Doxorubicin, Vincristine, Prednisone
RLN: Reactive lymph nodes
SCID: Severe combined immune deficiency
THL: Triple-hit Lymphoma
WHO: World Health Organization
XTT: Sodium 3′-[1-(phenylaminocarbonyl)-3,4-tetrazolium]-bis(4-methoxy-6-nitro) benzene sulfonic acid hydrate
