# Supplementary material for: Prohibitin (PHB) expression is associated with aggressiveness in DLBCL and flavagline-mediated inhibition of cytoplasmic PHB functions induces anti-tumor effects
Source: J Exp Clin Cancer Res. 2019 Nov 4;38:450. doi: 10.1186/s13046-019-1440-4 (PMC6830009; doi:10.1186/s13046-019-1440-4)
Supplement: Supplementary file 1 — Additional file 1. Supplementary materials. [file 13046_2019_1440_MOESM1_ESM.docx]

**Additional file 1**

**Supplemental Methods**

#### Immunofluorescence and confocal microscopy

AIF and PHB1 cellular (mitochondria, nucleus) localization in DLBCL cell lines upon FL3 exposure was studied by immunofluorescence staining and confocal microscopy analysis, as previously described (33). Primary antibodies used were: rabbit anti-AIF or anti-PHB1 (Cell Signaling Technology) and mouse anti-Tom20 (Santa Cruz Biotechnology) followed by incubation with Alexa-labeled secondary Abs (goat anti-mouse Alexafluor 594 or goat anti-rabbit Alexafluor 488, Invitrogen, Thermo Fisher Scientific) and finally with DAPI (Molecular Probes, Invitrogen). Images were captured using a Leica microscope and a Leica digital camera, and were processed using Leica IM500 Image Manager, or a confocal laser scanning microscope (LSM 510 META, Zeiss). The profiles of blue, green, and red fluorescence colocalization were analyzed using quantification software (Zeiss).

**Supplementary tables**

**Table S1. Correlation between PHB expression and clinic-biological parameters of patients with DLBCL.**

| Clinicopathological  Parameters  (N =82 patients) |  | PHB1  Low  (n = 33) | PHB1  High  (n = 49) | *P** Value | PHB2  Low  (n = 41) | PHB2  High  (n = 41) | *P** Value |
| --- | --- | --- | --- | --- | --- | --- | --- |
| Sex | Male  Female | 18  15 | 30  19 | *0.547* | 20  21 | 28  13 | *0.073* |
| Subtype | GCB  Non-GCB | 19  14 | 30  19 | *0.741* | 23  18 | 26  15 | *0.499* |
| LDH/NLDH | ≤ 1  > 1 | 21  12 | 20  29 | ***0.042*** | 24  17 | 17  24 | *0.122* |

***Chi-squared test**

**Table S2. Correlation between PHB expression and Ki-67 proliferation index.**

| *(N=73 patients)* | Ki-67 cut off | PHB1  Low  (n = 30) | PHB1  High  (n = 43) | *P**  Value | PHB2  Low  (n = 37) | PHB2  High  (n = 36) | *P** Value |
| --- | --- | --- | --- | --- | --- | --- | --- |
| Ki-67 | < 70%  ≥ 70% | 10  20 | 9  34 | *0.235* | 15  22 | 4  32 | ***0.004*** |
| Ki-67 | < 90%  ≥ 90% | 21  9 | 30  13 | *0.983* | 30  7 | 21  15 | ***0.034*** |

***Chi-squared test**

**Supplementary figures**

**Fig. S1 Confocal microscopy analysis of mitochondrial localization of PHB1 and AIF in GCB and ABC DLBCL cell lines under basal culture conditions.** SUDHL4 and OCI-LY3 were stained with anti-PHB1 or anti-AIF (green), anti-Tom20 (red, mitochondria) and DAPI (blue, nuclei). Merged images were performed and show PHB1 or AIF and mitochondria colocalizations (yellow) after 72 h of basal culture conditions. The profiles of blue, green, and red fluorescence colocalization were analyzed using quantification software (Zeiss). Representative data from at least three independent experiments illustrating PHB1 or AIF and Tom20 colocalization are shown; histograms display examples of measured fluorescence intensity along the line in the merge panels and peaks of fluorescence colocalization are labelled with arrows.

**Figure S2. FL3 determines the nuclear translocation of PHB1 in DLBCL cell lines.** The intracellular localization of PHB1was studied in SUDHL6 and OCI-LY3 cell lysate nuclear (N) and cytosol (C) fractions after 72 h of FL3 exposure by Western blot analysis. Histone H3 expression was used to demonstrate the purity of the fractions. Immunoblots have been quantified by densitometry and ratio of the quantitative values to total PHB1 expression has been incorporated below the western blot bands. Results are representative of two independent experiments.

**Figure S3. Analysis of PHB expression in DLBCL cell lysates after FL3 treatment. a** GCB (SUDHL4 and SUDH6) and ABC (OCI-LY3 and U2932) DLBCL cell lines were cultured for 24h with FL3 (20nM) or not (C, DMSO control). PHB1 and PHB2 expressions were evaluated by western blot in cell lysates. **b** Same results were obtained after 72h of FL3 (20nM) exposure and/or rituximab (RTX, 1µg/ml). Actin is shown as a loading control. Results are representative of 3 independent experiments.

 **a**

**b**

**Figure S4. FL3 reduces C-Raf phosphorylation in DLBCL cell lines.** GCB (SUDHL4, SUDHL6) and ABC (U2932, OCI-LY3) DLBCL cells were exposed 24h and 48h with FL3 (20nM). Then C-Raf phosphorylation status (on Ser289/296/301 or Ser338) was analyzed in cell lysates by Western blot. C-Raf (lower blot at 24h) was also analyzed when possible after stripping and re-probing western blot membranes. Actin is shown as a loading control. Results are representative of two independent experiments.

**Figure S5. Effect of FL3 on MNK1 protein and mRNA expression. a** Analysis of MNK1 protein levels determined by Western blotting after 72h treatment (FL3, 1-20 nM) as compared to control (C, DMSO control) in SUDHL4, SUDHL6 and OCI-LY3 cell lysates. **b** Relative quantification by qRT-PCR analysis of *MNK1* mRNA in presence or not of FL3 (20 nM). Results are expressed relative to control cultures as the means ± SD of three independent experiments. **: *p*<0.01 vs control.

**Figure S6. Effect of FL3 on PHB phosphorylation. a** Immunoprecipitation of GCB (SUDHL4, SUDHL6) and ABC (U2932, OCI-LY3, OCI-LY10) DLBCL untreated (C) or 24h FL3(20nM)-treated cell lysates, using Phospho-Akt Substrate (RXXS*/T*) Rabbit mAb (Sepharose® Bead Conjugate, Cell Signaling Technology). Akt phosphorylates substrates only at serine/threonine in a conserved motif characterized by arginine at positions -5 and -3 that are preferentially recognized by this antibody. Then, the eluted proteins were subjected to Western blot analysis using anti-PHB1 antibody (Cell Signaling Technology). **b** Total cell lysates following 24h exposure of FL3 (20 nM) were immunoprecipated using Dynabeads® Protein G incubated with anti phospho-(Ser/Thr)-Akt substrate primary antibody or anti-PHB1 primary antibody according to the manufacturer’s directions. Then PHB1 or phospho-(Ser/Thr)-Akt substrate expression respectively was analyzed in the immunocomplex by Western blot as shown for SUDHL4 and OCI-LY3 cell lysates. Input indicates nonimmunoprecipitated cell lysate (30µg of proteins); IgG, control IP with isotype antibody.


**Figure S7. Analysis of the apoptotic response induced by rituximab in DLBCL cell lines.** Flow cytometry analysis of rituximab-induced apoptosis was evaluated in the low CD20 positive cell line, U2932, as compared to the sensitive cell line, SUDHL4 using PI/Annexin V-FITC double staining. Cytogram data show representative results from one of three independent experiments of cells exposed or not (Control) to rituximab (1 µg/ml) for 72h. Apopotic (Annexin V–FITC^+^/PI^−^) cell percentages are done in the lower-right quadrant.
